# Chemerin sustains the growth of spongiotrophoblast and sinusoidal trophoblast giant cells through fatty acid oxidation

**DOI:** 10.1186/s12915-025-02294-9

**Published:** 2025-07-03

**Authors:** Jia xuan Cai, Ai wen Le, Xin yue Han, Jie Chen, Xiao hua Lei, Chen Huang, Jian V. Zhang

**Affiliations:** 1 Center for Energy Metabolism and Reproduction, Institute of Biomedicine and Biotechnology, Shenzhen Institute of Advanced Technology, Chinese Academy of Sciences, 1068 xueyuan Avenue, Shenzhen University Town, Guangdong, 518055 China; 2 Shenzhen College of Advanced Technology, University of Chinese Academy of Sciences, Shenzhen, China; 3 Department of Gynecology, Shenzhen Nanshan People’s Hospital, the Sixth Affiliated Hospital of Shenzhen University Health Science Center, Shenzhen, Guangdong China; 4 Department of Obstetrics and Gynecology, Shenzhen University General Hospital, Shenzhen, China; 5 Center for Energy Metabolism and Reproduction, Shenzhen Institute of Advanced Technology, Chinese Academy of Sciences, Shenzhen, China; 6 Faculty of Pharmaceutical Sciences, Shenzhen University of Advanced Technology, Shenzhen, China

**Keywords:** Chemerin, Lipid metabolism, Fatty acid oxidation, Spongiotrophoblast cell, Sinusoidal trophoblast giant cells, Glycogen trophoblast cells

## Abstract

**Background:**

Placental metabolic dysfunction is associated with pregnancy complications, including preeclampsia, gestational diabetes mellitus, and fetal growth restriction. However, little is known about how the metabolic processes regulate placental development and trophoblast differentiation. The adipokine chemerin, whose serum level is elevated during pregnancy, regulates the placental lipid metabolism and may influence placental development and trophoblast differentiation.

**Results:**

In this study, we observed the increased chemerin expression in the serum and placenta of the pregnant mice. Chemerin is highly expressed in the extraembryonic primary parietal trophoblast giant cells and the ectoplacental cone (EPC) trophoblast cells. Excessive chemerin treatment in mice results in increased placental lipid accumulation, promotes the expansion of glycogen trophoblast cell (GlyT) and syncytiotrophoblast, and restricts the growth of spongiotrophoblast (SpT) and sinusoidal trophoblast giant cell (S-TGC). Chemerin deficiency led to increased expression of placental fatty acid oxidation enzymes and disrupted the proliferation of SpT and S-TGC in the labyrinth. Furthermore, we utilized the fatty acid oxidation inhibitor etomoxir and demonstrated that blocking fatty acid oxidation hinders the proliferation of SpT and S-TGC in the labyrinth.

**Conclusions:**

Our study demonstrated that chemerin-related lipid metabolism is crucial for EPC trophoblast differentiation during placental development, providing evidence that chemerin determines the growth of SpT and S-TGC through fatty acid oxidation. These findings also imply a possible pathological mechanism for pregnancy complications associated with chemerin.

**Supplementary Information:**

The online version contains supplementary material available at 10.1186/s12915-025-02294-9.

## Background

During pregnancy, the placenta plays a crucial role in mediating the exchange of nutrients and gases between the mother and fetus, producing hormones, and serving as a maternal–fetal interface to ensure the embryo's adequate development [1, 2]. To support these processes, the placenta has an extraordinarily high metabolic rate, consuming approximately 40% of the oxygen of the entire conceptus [3]. Despite the placental metabolic processes remaining largely unknown, the abnormalities of placental metabolism have been associated with the occurrence of pregnancy complications, including preeclampsia [4], gestational diabetes mellitus [5], and fetal growth restriction [6, 7]. Recent isotope tracing and compartmental metabolomics data from the mouse placenta indicate that maternal glucose circulation is rapidly transferred to the embryo during mid-gestation, with significantly lower enrichments in the placenta [8, 9]. Other metabolic processes may be involved in sustaining the placenta during a tenfold increase in tissue mass. Fatty acids exhibit increased serum levels during pregnancy [10], and the enzymes involved in fatty acid oxidation are active in the placenta [11], indicating that fatty acids serve as a primary source of energy for placental development.

Chemerin is an adipokine primarily secreted by white adipose tissue [12, 13], regulating various physiological functions, including lipid metabolism, inflammation, and innate immunity [12, 14–16]. Chemerin levels are elevated in multiple metabolic diseases, including obesity, insulin resistance, and type 2 diabetes [17–19]. During pregnancy, circulating chemerin levels in the serum increase continuously throughout gestation and then decline rapidly after delivery. Excessive chemerin promotes M1 macrophage activation [20] and increases inflammatory cytokine levels in the placenta, leading to trophoblast apoptosis and symptoms of preeclampsia [21, 22]. Elevated chemerin concentrations accelerate placental lipid accumulation and contribute to glucose intolerance in mice with high-fat diet-induced gestational diabetes mellitus (GDM)[23]. These studies indicate that high chemerin levels play a significant role in adverse pregnancy outcomes, such as preeclampsia and gestational diabetes mellitus [24, 25]. However, the role of chemerin in placental development and trophoblast differentiation remains unclear.

In this study, we examined chemerin expression in the serum and placenta throughout the process of mouse pregnancy. We conducted experiments with exogenous chemerin-treated mice and chemerin knockout mice to investigate the effect of chemerin on placental lipid metabolism and trophoblast differentiation. We used etomoxir-treated mice to demonstrate the role of fatty acid oxidation in the SpT and S-TGC. Overall, this study highlighted the critical role of chemerin in the differentiation of ectoplacental cone progenitor lineages and provided evidence that chemerin influences the differentiation of SpT and S-TGC by regulating fatty acid oxidation.

## Results

### Chemerin exhibits dynamic expression in the serum and placenta of pregnant mice

**Fig. 1:**
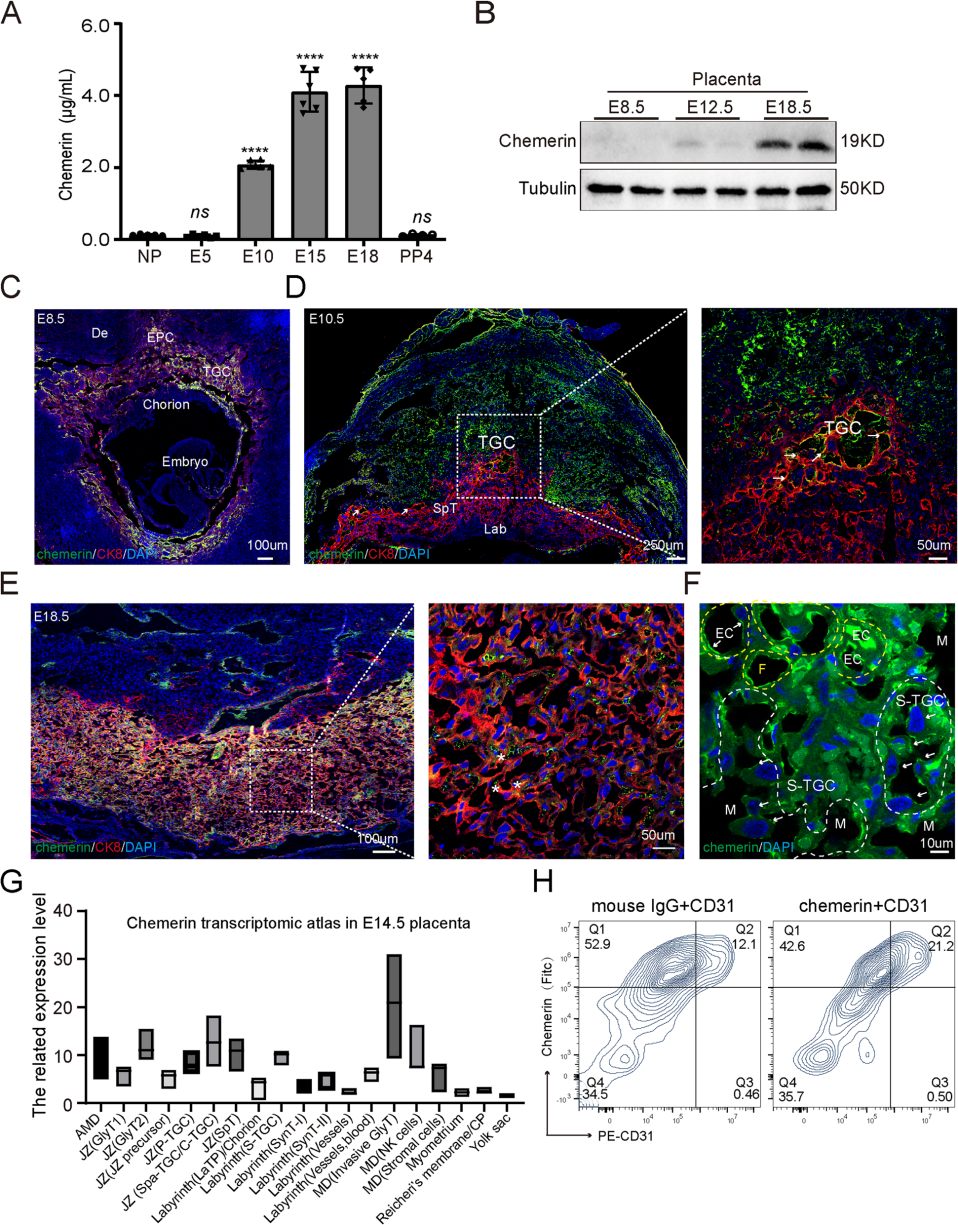
The dynamic expression of chemerin during mouse placental development. **A** Chemerin concentration on different mouse gestational days (NP, non-pregnancy, *n* = 5; E5, *n* = 6; E10, *n* = 6; E15, *n* = 6; E18, *n* = 5; PP4, post-pregnancy4, *n* = 4). ****p < 0.0001 compared to NP using one-way ANOVA and Tukey’s test. The graphs depict mean ± SEM values. **B** The protein expression of chemerin on different days of the mouse placenta. **C**–**E** Chemerin expression (green) was examined on E8.5 (**C**), 10.5 (**D**), and 18.5 (**E**) placentas with the trophoblast marker cytokeratin 8 (CK8, red). Representative staining images show chemerin signal accumulation on the ectoplacental cone (EPC) and trophoblast giant cell (TGC) but little expression on the chorion and embryo. De indicates decidua. The control staining with mouse IgG and rat IgG on E8.5, 10.5, and 18.5 is shown in Additional file 1: Fig. S1A, B. **D** The square frame image shows chemerin expression at the boundary of the maternal–fetal interface at E10.5 (arrow indicates a representative staining area). Trophoblast giant cell (TGC), spongiotrophoblast (SpT), labyrinthine (Lab). **E**, **F** Representative staining of E18.5 placentas, as indicated by the smaller square frame in the labyrinthine region, revealed chemerin distribution on syncytiotrophoblast and fetal endothelial cells. Yellow F represents the fetal side, and white M represents the maternal side, which is outlined with a dotted line. The arrow indicates chemerin staining on the sinusoidal TGC on the maternal side and the endothelial cells on the fetal side. **G** The boxplot describes the related mean expression of chemerin in two E14.5 placenta sections from the publicly spatiotemporal transcriptomic data (*n* = 2). The spatial expression data can be downloaded from the website: https://db.cngb.org/stomics/mpsta/. AMD (anti-myometrial decidua), GlyT (glycogen trophoblast), MD (myometrial decidua), and CP chorionic plate. **H** Flow cytometry analysis of chemerin expression in fetal endothelial cells (PE-CD31-positive) at E18.5 wild-type placenta. The quadrant of Q2 in the graph indicates that chemerin exhibited a higher expression (21.2%) in fetal endothelial cells compared to the IgG-labeled group (12.1%). The WB and staining array were repeated 3–4 times for confirmation. The individual values in chemerin concentration (**A**) and mean expression of chemerin at E14.5 placenta (**G**) were showed in Additional file 5

To investigate the role of chemerin during pregnancy, we first measured chemerin serum levels on different days of pregnancy in mice. The results showed that serum chemerin levels were significantly elevated from E10, remained at this level until the end of gestation, and then declined rapidly after birth (Fig. 1A). Consistent with this, chemerin showed increased protein expression with placental development in mice (Fig. 1B), indicating that chemerin has a physiological effect on placental development.

We then sought to verify the expression and localization of chemerin in mouse placentation. Through immunostaining of E8.5 mouse placenta, we observed that most chemerin is expressed on the extraembryonic trophoblast (Fig. 1C) compared to mouse IgG (chemerin) and rat IgG (CK8) staining (Additional file1: Fig. S1A, B). The co-staining image with the trophoblast-specific marker cytokeratin (CK8) revealed that chemerin was primarily localized on the ectoplacental cone (EPC) and the adjacent primary trophoblast giant cell (P-TGC), with minimal expression on the chorion plate and embryo. At E10.5, most chemerin accumulated on the decidua and trophoblast giant cells between the junctional zone and decidua (Fig. 1D). Along with the gestational process, chemerin was mainly expressed in the labyrinthine and junction zone but seems to have little expression on syncytiotrophoblast when evaluated with CK8 staining on E18.5 placenta (Fig. 1E). The smaller version in the labyrinth showed that chemerin mainly accumulated on sinusoid trophoblast giant cells (S-TGC) of the maternal side and endothelial cells on the fetal blood side (Fig. 1F). The published spatiotemporal transcriptomic atlas data [26] demonstrated that chemerin was highly expressed in the EPC and P-TGC of the E8.5 uterine section (Additional file1:Fig. S2), and the trophoblast giant cells of the junction zone and S-TGC of the labyrinth in the E14.5 placenta (Fig. 1G). Meanwhile, the cytometry results from the E18.5 placenta further confirmed that chemerin was positively expressed in the fetal endothelial cells (Fig. 1H). These expression results suggest that chemerin has a dynamic expression during placenta development and may directly influence placenta development.

### Excessive chemerin facilitates placental lipid accumulation and impairs placental development

**Fig. 2:**
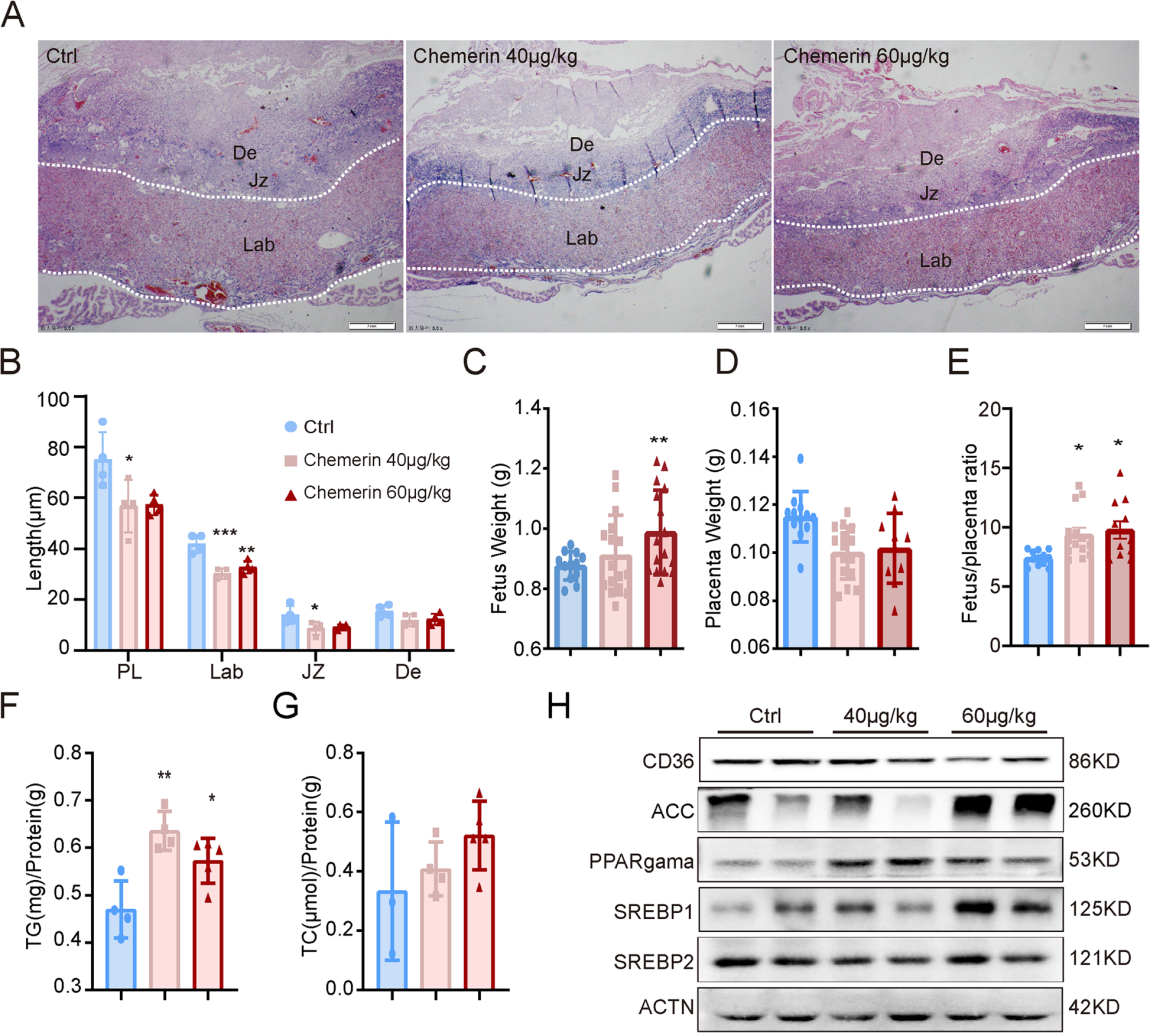
Excessive chemerin impairs placental development and lipid metabolism. **A** Representative image of E18.5 placentas after chemerin was administered intravenously on E14.5 and E16.5. Lab, labyrinthine; Jz, junctional zone; De, decidua. Chemerin-treated concentrations of 40 µg/kg and 60 µg/kg, with each group consisting of four mice. **B** The lengths of the placenta, labyrinth, junction zone, and decidua were measured and quantified using ImageJ (qualification data from 4 mice in each group). *p < 0.05, ** *p* < 0.01, and ***p < 0.001 compared to the control group. **C**–**E** Fetus weight, placenta weight, and fetus/placenta ratio are measured on E18.5. *p < 0.05, **p < 0.01 compared to Ctrl group (Ctrl, *n* = 12; Chem40, *n* = 15; Chem60, *n* = 10). **F**–**G** The concentrations of TG (triglyceride) and TC (cholesterol) on placenta. * *p* < 0.05, **p < 0.01 compared with the control group (Ctrl, *n* = 4 mice; Chem40, *n* = 4 mice; Chem60, *n* = 5 mice). **H** The expression of lipid metabolism-related genes, including CD36, ACC1, PPARγ, SREBP1, and SREBP2, was measured in the placentas and repeated three times. The graphs depict mean ± SEM values and analysis with one-way ANOVA and Tukey’s test. The individual values of the lengths of placentas (**B**) and the concentration of TG (**F**) and TC (**G**) were shown in additional file 5.

To investigate the role of chemerin in the placenta, we treated C57BL/6 J pregnant mice with 40 µg/kg and 60 µg/kg recombinant chemerin peptide through intravenous injection. We injected the mice on E14.5 and E16.5 and assessed the pregnancy outcome on E18.5. Chemerin treatment did not trigger systemic toxicity and demise of the fetus, as mouse absorption or embryo resorption was not observed. Chemerin impaired placental development as placenta length (PL) and labyrinthine length (Lab) were distinctly smaller than those of the control mice (Fig. 2A, B), which resulted in decreased placental weight, increased fetal weight, and fetal/placenta ratio in the chemerin-treated mice (Fig. 2C–E).

Further, we measured placental triglyceride (TG) and total cholesterol (TC) levels. As expected, chemerin treatment significantly elevates TG content (Fig. 2F, G), facilitating lipid droplet accumulation on the placenta, as indicated by oil red O staining results (Additional file1:Fig. S3A), and increasing the expression of lipid key-regulated genes PPARγ, SREBP1, and the fatty acid synthesis rate-limiting enzyme acetyl-CoA carboxylase (ACC) (Fig. 2H). Additionally, we found that chemerin treatment decreased the lipid content of gonadal fat tissue, as evidenced by smaller adipocyte size after treatment (Additional file1:Fig. S3B). These data suggest that chemerin facilitates lipid accumulation in the placenta, and excessive chemerin impairs placental development.

### Deficiency of chemerin compromises the abnormal growth of SpT and S-TGC

**Fig. 3:**
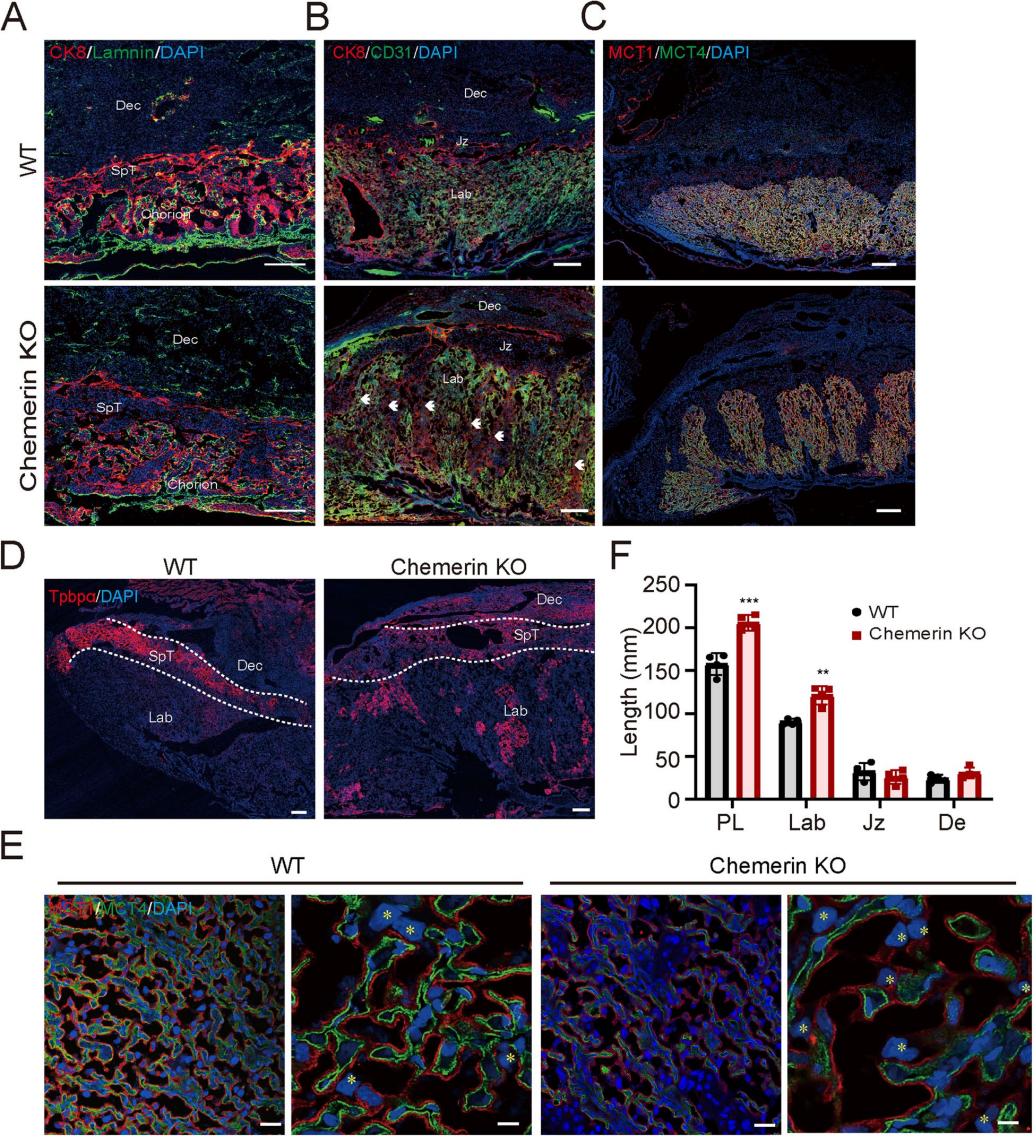
Loss of chemerin results in the abnormal of placenta. **A**–**E** WT and chemerin knockout placentas phenotype examination. **A** E10.5 placentas with CK8 and laminin staining in WT and chemerin KO placentas. Laminin, a marker of fetal blood endothelial cells (green), and CK8, a marker of trophoblasts (red). The control staining in E10.5 and 18.5 was shown in Additional file 1: Fig. S1C, D. **B**, **D** The staining with CK8 and CD31 (**B**), MCT1 and MCT4 (**C**), and TPBPα (**D**) in the E18.5 WT and chemerin KO placentas (scale bar = 200 µm). **E** The MCT1 and MCT4 staining was performed in the E18.5 WT and KO placentas (scale bar = 40 µm) and magnified with the representative image (scale bar = 10 µm). Yellow * indicates the number of sinuses TGCs on the maternal blood size in the image. All staining assays were performed three to four times. **F** The comparison of the length of the placenta (PL), labyrinthine (Lab), junctional zone (Jz), and decidua (De) between wild-type (WT) mice (*n* = 4) and chemerin knockout (KO) mice (*n* = 4), ***p < 0.001, **p < 0.01 compared to the WT group. Graphs depict the mean ± SEM and analysis for each group, with a non-paired two-tailed *t*-test used for comparison. The individual values of the length of placentas were shown in Additional file 5

To further determine chemerin’s role in the placenta, we examined the placenta phenotype in chemerin knockout (chemerin KO) mice and wild-type mice after confirming the global loss of chemerin expression in KO mice (Additional file1:Fig. S4A). Chemerin KO placenta at E10.5 exhibited apparent junctional zone formation compared to the WT placenta, which was deeply intermingled into the labyrinth (Fig. 3A and rabbit IgG staining in Additional file1:Fig. S1C, D). At E18.5, the chemerin KO placenta showed fetal vessel loss in the labyrinth compared with the uniform distribution of trophoblast and fetal vessels in the WT placenta (Fig. 3B). These branched trophoblasts were not derived from the syncytiotrophoblast in the labyrinth when evaluated with MCT1 and MCT4 (Fig. 3C), but originated from Tpbpα positive expressed SpT and glycogen trophoblast cells (GlyT) of the junction zone (Fig. 3D). Further staining with periodic acid-Schiff (PAS) showed that these TPBPα-positive trophoblasts were more likely attributed to SpT, although some clusters of GlyT also emerged in the labyrinth (Additional file1: Fig. S4B). Loss of chemerin in the placenta also increased S-TGC cell numbers and the labyrinth area (Fig. 3E, F and Additional file1: Fig.S4C) and restricted fetal growth (Additional file1:Fig. S4D, E). These results indicate that chemerin deficiency leads to the abnormal growth of EPC progenitor-derived SpT and S-TGC, ultimately resulting in aberrant placental development and restricted fetal growth.

### Lack of chemerin leads to abnormal lipid metabolism in the placenta

**Fig. 4:**
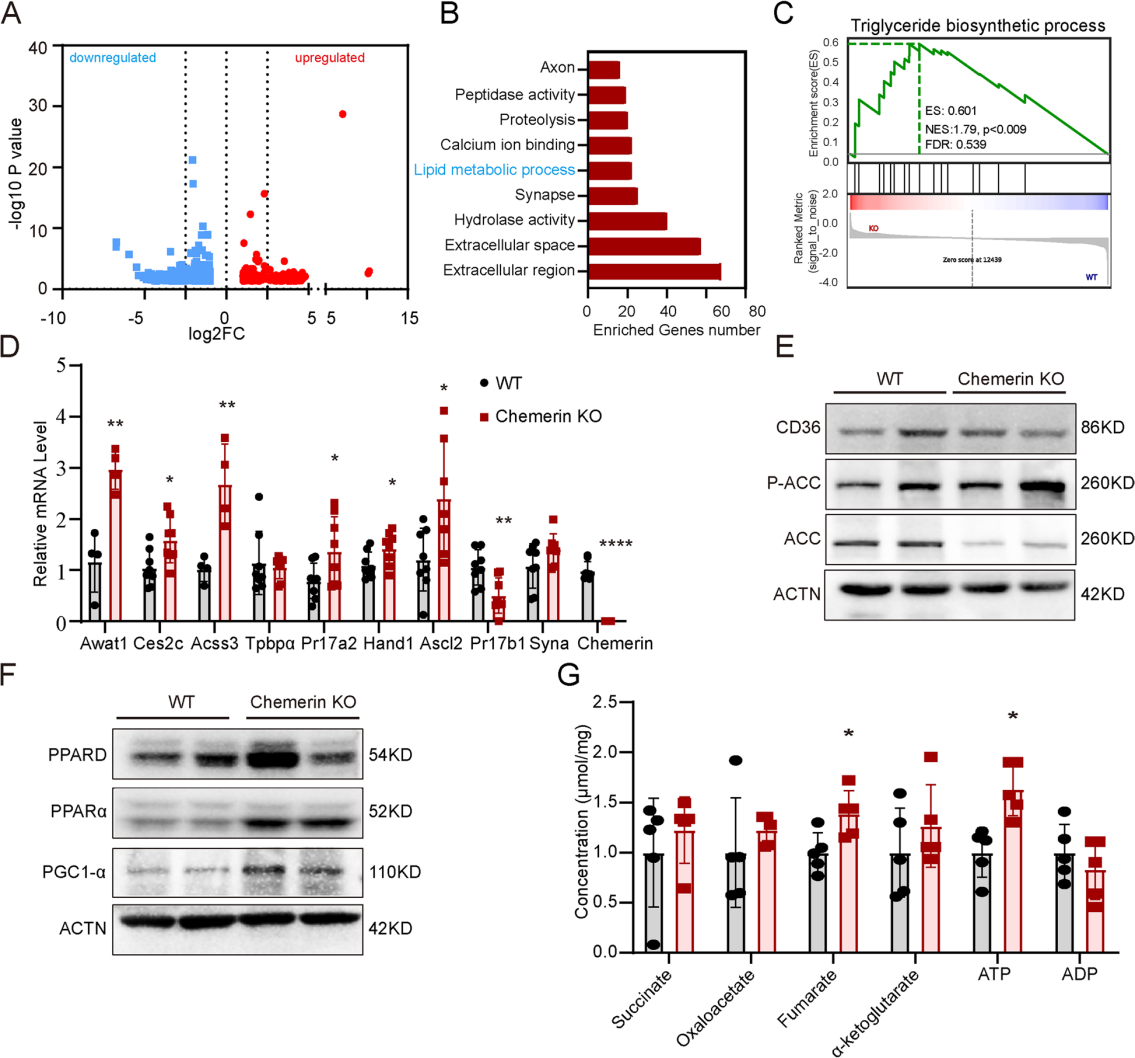
Loss of chemerin results in dysregulated lipid metabolism in the placenta. **A** Volcano plot showing the differentially expressed genes in the placenta of wild-type (WT) mice (*n* = 4) and chemerin knockout (KO) mice (*n* = 4). Blue dots represent significantly down-regulated transcripts with a *P*-value of ≤ 0.05 and a log2-fold change of < − 2. Red dots represent significantly upregulated transcripts with a *P*-value of ≤ 0.05 and a log2-fold change of ≥ 2. **B** The bar graph shows the enriched top 10 biological pathways from significantly changed genes in the chemerin KO mouse placenta. **C** Enrichment plots of selected gene sets from GSEA were enriched in the chemerin KO placenta. **D** qRT-PCR validates the lipid metabolism enriched transcripts expression (Awat1, Ces2c, Acss3) and SpT (Tpbpα, Pr17a2), S-TGC (Hand1, Ascl2), GlyT (Pr17b1) syncytiotrophoblast (Synα) specific transcripts from WT (*n* = 4) and chemerin KO (*n* = 4) placentas. *p < 0.05; **p < 0.01. This assay is repeated three times and analyzed with a non-paired two-tailed *t*-test. **E**, **F** The expression of fatty acid uptake, synthesis (**E**), and oxidation metabolism-related enzymes (**F**) was measured in WT and chemerin KO placenta and repeated three times. **G** The intermediate metabolites of the oxidative phosphorylation process contain a value on chemerin KO (*n* = 5 mice) placenta compared to WT (*n* = 5 mice) placenta. * *p* < 0.05 as determined by a non-paired *t*-test. All graphs depict mean ± SEM values. The individual values for RNA expression (**D**) and intermediate metabolites were shown in Additional file 5

To investigate the deregulated molecular pathway in the chemerin knockout (KO) placenta, we compared the transcriptomic profiles of wild-type (WT) and chemerin KO placental tissue at E18.5 using RNA sequencing technology. There were 521 transcripts altered by chemerin deficiency, including 314 genes downregulated and 207 genes upregulated (false detection rate-corrected P < 0.05 and log2FC > 1; Fig. 4A and Additional file 2). Gene Ontology (GO) analysis identified the lipid metabolism process, along with extracellular region and space, hydrolase activity, and synapse as the top 10 altered pathways in the chemerin knockout placenta (Fig. 4B). GSEA analysis showed that the triglyceride biosynthetic process was enriched in the chemerin knockout placenta (Fig. 4C). Quantitative PCR results showed that the transcripts related to lipid metabolism, including Awat1 (esterify long chain (wax) alcohols with acyl-CoA-derived fatty acids to produce wax esters) [27], *Ces2c* (hydrolyze endogenous lipid substrates such as TGs and diglycerides) [28], and *Acess3* (mitochondrial production of acetyol-CoA) [29], were all upregulated in chemerin KO placenta (Fig. 4D). This result also confirmed that the lack of chemerin indeed increased gene expression of SpT (Prl7a2, Ascl2) and S-TGC (Hand1, Ascl2) and decreased expression in GlyT (Prl7b1) but had no effect on syncytiotrophoblast (Syna) in the placenta (Fig. 4D).

Furthermore, we examined the expression of lipid metabolism enzymes in WT and chemerin KO placentas. The protein expression results showed that a deficiency of chemerin did not alter placental lipid uptake (CD36), but reduced fatty acid synthesis (ACC1 and P-ACC1) and increased fatty acid oxidation metabolism (PPARD, PPARα, PGC1-α) (Fig. 4E, F). Consistent with oxidation enzyme expression, the chemerin KO placenta had increased NAD-retinol dehydrogenase activity (Additional file1:Fig.S5A) and reduced NADH level (Additional file1:Fig. S5B). More importantly, the intermediate metabolites of the oxidative phosphorylation process, fumarate and ATP, were elevated in the chemerin KO placenta (Fig. 4G and Additional file3). These results suggest that the loss of chemerin impairs placental lipid synthesis and increases fatty acid oxidation activity.

### Disruption of fatty acid oxidation impairs SpT and S-TGC growth in chemerin KO placenta

**Fig. 5:**
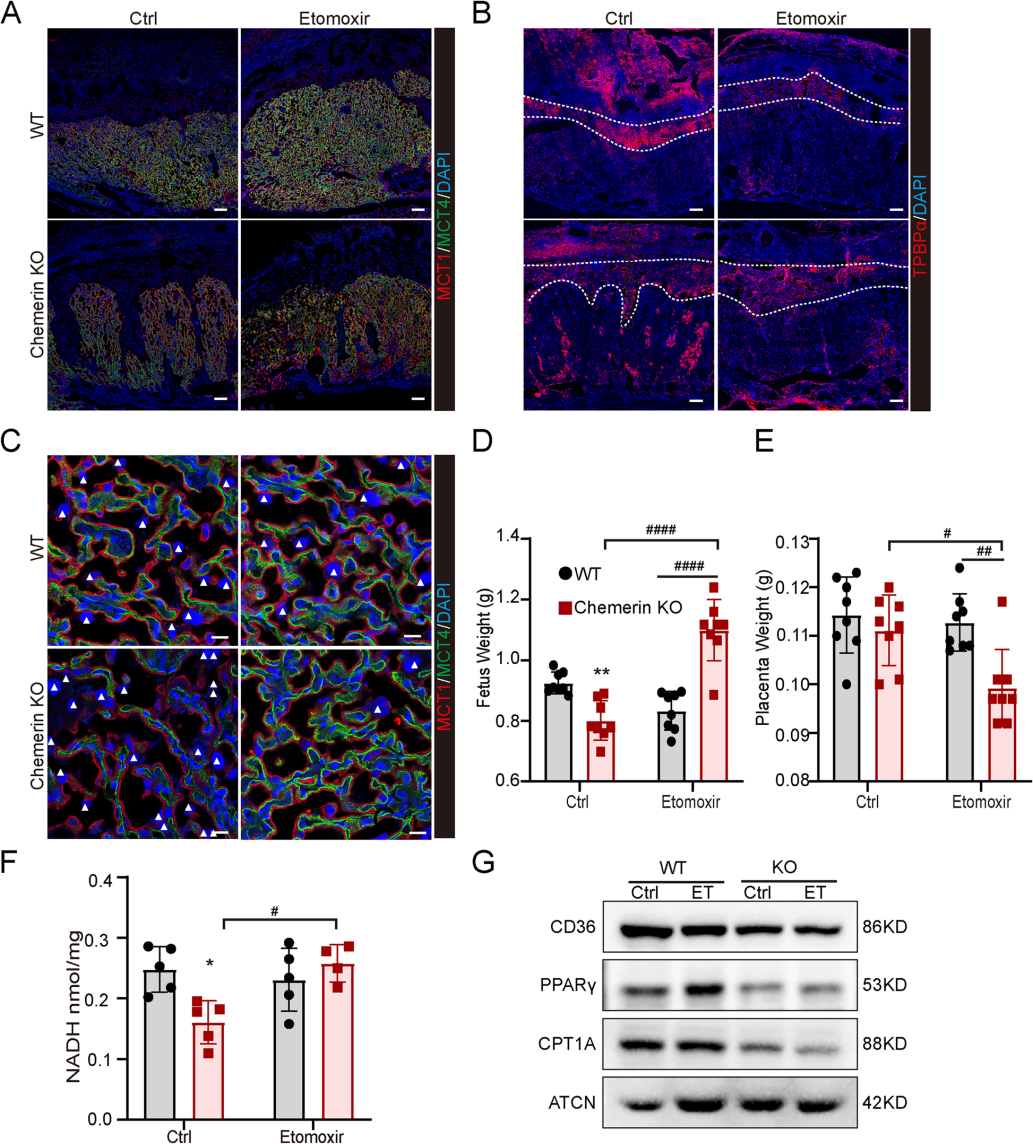
Inhabitation fatty acid oxidation impairs SpT and S-TGC growth in chemerin KO placenta. **A** The staining images of MCT1 (red) and MCT4 (green) were representatively shown in the 20 mg/kg etomoxir-treated WT and chemerin KO placentas. Scale bar = 200 µm. **B** The image staining with Tpbpα examined the etomoxir effect on SpT growth on WT and KO placenta. Scale bar = 200 µm. **C** Representative images of MCT1 and MCT4 staining on WT and KO placenta. Scale bar = 20 µm. The white triangle arrowhead pointed out the number of S-TGC on the maternal side. All staining assays are performed three to four times for detection. **D**, **E** The fetal weight (**D**) and placental weight (**E**) of WT and chemerin KO mice after etomoxir treatment. **p < 0.01 compared with WT ctrl group, #*p* < 0.05, ###*p* < 0.001, ####*p* < 0.0001 compared with KO ctrl group (WT-ctrl *n* = 8; WT-ET, *n* = 8; KO-ctrl, *n* = 8; KO-ET, *n* = 8). **F** The concentration of NADH in placentas from WT and chemerin KO mice after etomoxir treatment (WT-ctrl *n* = 5; WT-ET, *n* = 5; KO-ctrl, *n* = 5; KO-ET, *n* = 4). *p < 0.05 compared with WT ctrl group, #*p* < 0.05 compared with KO ctrl group. **G** The expression of fatty acid metabolism genes, including CD36, PPARγ, and CPT1A, in WT and KO placenta. ET as an etomoxir-treated group. All graphs depict mean ± SEM values, with statistical analysis performed using two-way ANOVA and Tukey's post hoc test. The individual values for fetal weight (**D**), placental weight (**E**), and the concentration of NADH (**F**) were shown in Additional file 5

Due to chemerin’s effect on placental fatty acid oxidation and trophoblasts, SpT and S-TGC, we treated WT and chemerin KO mice with the fatty acid oxidation inhibitor etomoxir (ET) to examine the placental phenotype. After being treated with 20 mg/kg ET at E14.5 and E16.5, the placental crossing staining results showed ET treatment ameliorated chemerin knockout-induced abnormalities of the syncytiotrophoblast branch (Fig. 5A), repressed the clusters of SpT in the labyrinth (Fig. 5B), and eliminated S-TGCs on both WT and chemerin KO placentas (Fig. 5C). Etomoxir treatment also increased chemerin KO placental fetal weight and reduced placental weight (Fig. 5D, E). Consistently, etomoxir elevated the NADH level in chemerin KO placenta (Fig. 5F) and reduced the expression of key lipid and fatty acid oxidation enzymes, including PPARγ and carnitine palmitoyl-transferase 1 isoform (CPT1A), in the placenta (Fig. 5G). These results suggest that the placenta utilizes the energy of fatty acid oxidation to sustain SpT and S-TGC growth, which further impacts the syncytiotrophoblast branch and fetal development.

### Excessive chemerin suppresses the growth of SpT and S-TGCs in the placenta

**Fig. 6:**
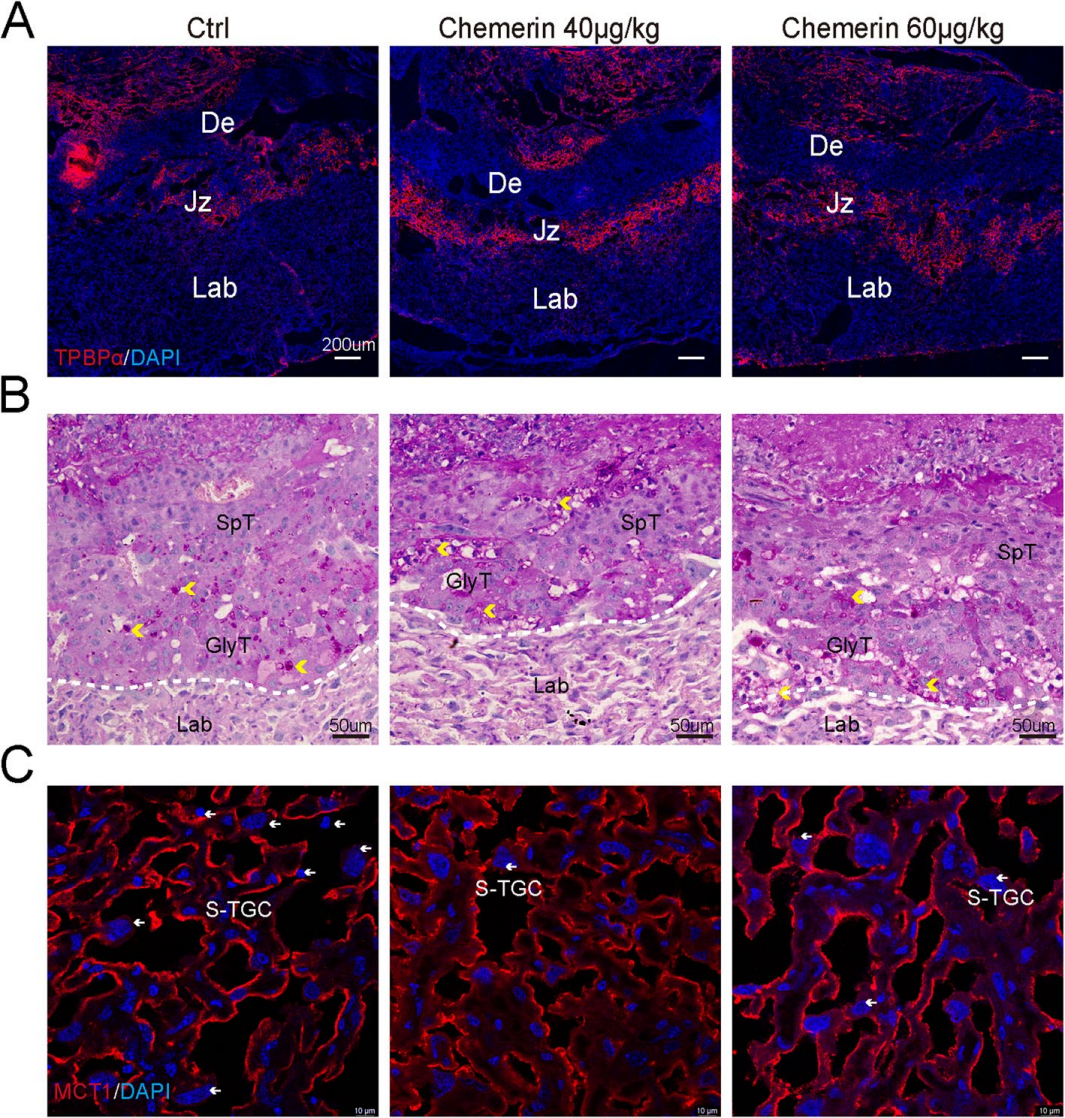
Excessive chemerin suppresses SpT and S-TGC growth in the placenta. **A** Representative image of GD18.8 placentas with immunostaining of Tpbpα (red) in chemerin-treated mice. Scale bar = 200 µm. **B** The image of PAS staining on the chemerin-treated placenta. Scale bar = 50 µm. Yellow arrowheads indicate the GlyT in the junctional zone. **C** MCT1 staining on chemerin-treated placentas reveals the phenotypes of syncytiotrophoblast and S-TGC in the labyrinth. The white arrow labels the S-TGC adjacent to the syncytiotrophoblast. These staining assays were repeated 2–3 times on chemerin-treated mice (control mice, *n* = 4; chemerin 40 µg/kg, *n* = 4; chemerin 60 µg/kg, *n* = 4)

Since the deficiency of chemerin promotes SpT and S-TGC growth, we examined the effect of chemerin peptide on these trophoblast cells. Chemerin treatment did not alter Tpbpα expression in the placentas (Fig. 6A), increased GlyT accumulation, and restricted SpT growth as evaluated by PAS staining (Fig. 6B). Meanwhile, chemerin peptide expanded the growth of syncytia and suppressed S-TGC growth in the labyrinth (Fig. 6C). These results confirmed that chemerin-induced lipid synthesis limits the growth of SpT and S-TGC.

### Trophoblast-derived chemerin has a similar role with chemerin KO placenta

**Fig. 7:**
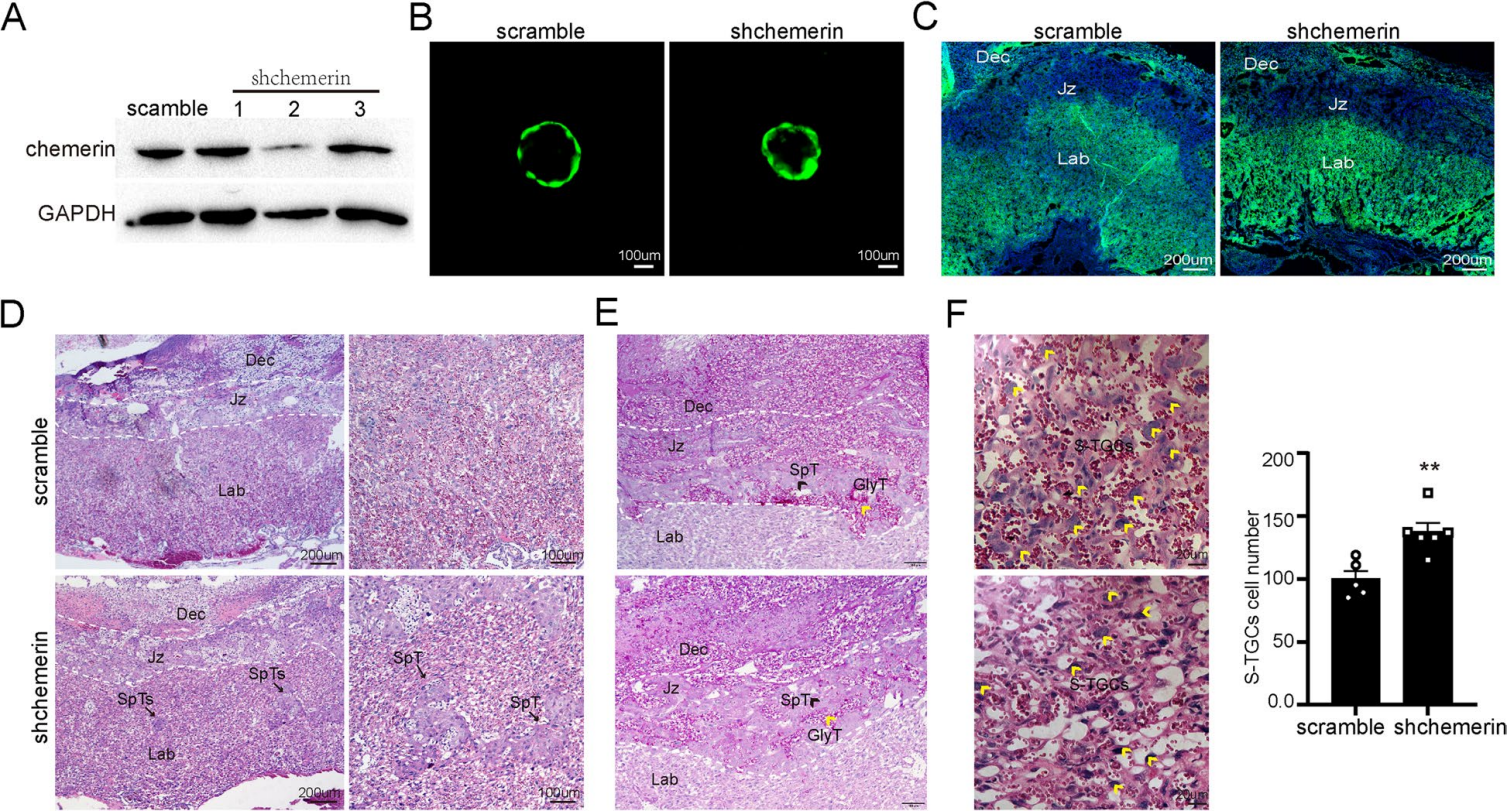
Trophoblast-derived chemerin has a determined role in SpT and S-TGC growth. **A** The expression of chemerin was determined in MS1 cells after lentiviral-mediated infection with scramble shRNA and three chemerin-targeted shRNA. WB results showed that chemerin shRNA2 had significant suppression of chemerin. The following experiments utilized the chemerin shRNA2 as the chemerin target sequence. **B** Blastocysts transduced with lentiviruses expressing scramble shRNA (scramble) or chemerin shRNA2 (shchemerin) showed GFP expression in the trophectoderm but not in the inner cell mass. **C** At E18.5, GFP expression was observed in the junction zones and labyrinth layers of the scramble and shchemerin group placenta. **D**, **E** HE (**D**) and PAS (**E**) staining were used to compare the scramble and shchemerin placenta phenotypes. The blank arrow indicated that SpT was intermingled within the labyrinth of the shchemerin group. **F** The smaller version of the labyrinth showed the structure of maternal and fetal sinusoids. The cell numbers of S-TGC on the maternal side were quantified in the scramble (*n* = 5) and shchemerin placentas (*n* = 7). The S-TGC cell numbers from each group, obtained from three mice, were compared using non-paired two-tailed *t*-tests. **p < 0.01 and ****p < 0.0001 compared with the WT group using a non-paired two-tailed *t*-test. The individual values of S-TGC numbers were shown in Additional file 5

Although chemerin’s role in SpT and S-TGC has been demonstrated, the different origin of chemerin in the placenta needs to be further determined. We constructed an effective lentiviral shRNA targeting chemerin (Fig. 7A) and packaged the scramble and shRNA viruses to generate chemerin trophoblast-specific knockdown placenta through the infection of blastocysts (Fig. 7B). The GFP expression carried by the lentiviral vector was examined in the junctional zone and labyrinth trophoblast in both scramble and chemerin shRNA-infected placenta (Fig. 7C). The chemerin shRNA-infected placenta indeed had intermingled SpT clusters in the labyrinth, and the increased cell number of S-TGC, compared to the scramble-infected placenta (Fig. 7D–F). Despite the placenta in the chemerin shRNA group exhibiting a similar phenotype to that of chemerin knockout mice, the effect on SpT and S-TGC was significantly lower than in knockout mice, suggesting that maternal-derived chemerin also affects placental development.

## Discussion

**Fig. 8:**
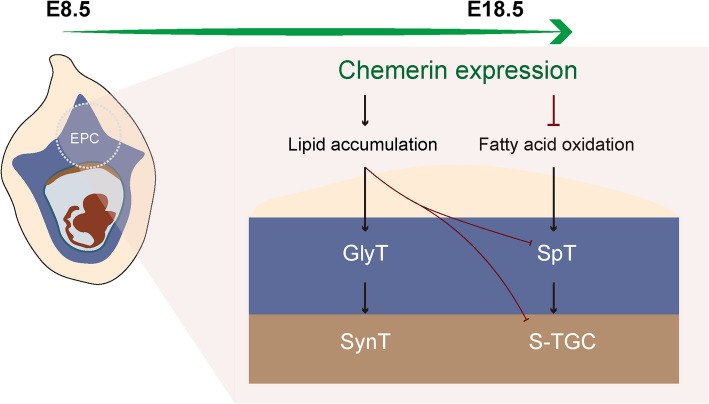
Schematic representation of the physiological role of chemerin in the mouse placenta. This graphic depicts the role of chemerin in regulating mouse trophoblast lineage differentiation through lipid metabolism. Along with the placental development from E8.5 to E18.5, chemerin expression increases placentas lipid accumulation, which facilitates glycogen accumulation on GlyT and the expansion of syncytiotrophoblast but limits the cell growth of SpT in the junction zone and S-TGC in the labyrinth. The lack of chemerin impaires placental fatty acid oxidation, consequently leads to the overgrowth of SpT and S-TGC. This study examines the physiological function of chemerin in mouse placental development, emphasizing the role of lipid metabolism, specifically fatty acid oxidation, in the differentiation of ectoplacental cone progenitor cell lineages

Placental development is a tightly controlled event that relies on adequate regulation of trophoblast cell differentiation. Here, our results found that the adipokine chemerin is expressed in the ectoplacental cone and plays a crucial role in the growth of ectoplacental cone-derived trophoblasts. The deficiency of chemerin results in the expansion of the spongiotrophoblast (SpT) and sinusoid trophoblast giant cells (S-TGC) in the placenta, whereas excessive chemerin limits the cell growth of the SpT and S-TGC, facilitating the expansion of the GlyT and syncytiotrophoblast. Secondly, we provided evidence that chemerin regulates EPC-derived trophoblast growth through lipid metabolism. Chemerin increases placental lipid accumulation, elevates the expression of fatty acid synthesis enzymes, and promotes glycogen accumulation in the GlyT and syncytiotrophoblast, thereby facilitating their expansion. The deficiency of chemerin enforces placental fatty acid flux to the oxidation catabolism pathway and the outgrowth of SpT and S-TGC (Fig. 8). These results suggest that fatty acids in the placenta are not only transported to the fetus but are also involved in the lineage differentiation of EPC progenitors. Abnormal chemerin expression disturbs placental lipid metabolism and trophoblast differentiation, which could imply a potential pathological mechanism for chemerin-related human pregnancy complications.

This study demonstrated the role of fatty acid oxidation on SpT and S-TGC growth. These two trophoblast cells secrete placental hormones, playing an endocrine role in sustaining both maternal and placental function for a successful pregnancy [30, 31]. A previous study reported that fatty acid oxidation enzymes are substantially expressed in placental villi, where trophoblast exhibits comparable fatty acid oxidation activity to that of skeletal muscle and fibroblasts, enabling it to satisfy high energy requirements [11]. In other group studies, decreased fatty acid oxidation gene expression was observed in preeclampsia placentas, and the degree of reduction was related to severe and early-onset preeclampsia [11, 32, 33]. In contrast, the placentas of obese women show reduced activity of fatty acid oxidation and accumulated lipids in early pregnancy [34], leading to the accumulation of oxidized metabolites for mother and baby. These findings suggest that fatty acid oxidation metabolism supports the growth of mouse placenta and potentially plays a role in human trophoblast function.

Aberrant spongiotrophoblast differentiation has been previously reported in diabetic placentae, a model induced by streptozotocin. Diabetic conditions lead to ectopic proliferation of spongiotrophoblast-like cells in the labyrinthine layer, a reduced junctional zone, and a smaller labyrinth and fetal size [35]*.* The phenotypes are very similar to those of chemerin knockout mice, even though the chemerin knockout placenta exhibits an expansion of the labyrinth area, which is more likely due to the loosening of the maternal and fetal vascular structures. Besides regulating spongiotrophoblast differentiation, chemerin is expressed on trophoblast giant cells of the E10.5 placenta, which originate from Tpbpα progenitors in the junctional zone and are considered analogous to the extra-villous trophoblast (EVT) of the human placenta [36]. The extra-villous trophoblast is crucial for remodeling the spiral arteries during mid-gestation. Insufficient EVT invasion and vascular remodeling contribute to pregnancy disorders like preeclampsia, preterm birth, and intrauterine growth restriction. Chemerin had increased serum levels in preeclampsia and has been considered a biomarker of preeclampsia in some studies [37]. Hence, it is necessary to investigate further the role of chemerin in human trophoblast differentiation and trophoblast cell invasion to uncover the pathological effects of these pregnancy disorders.

## Conclusions

In summary, this study describes the role of chemerin in the differentiation of ectoplacental cone progenitor lineages and provides evidence that chemerin determines SpT and S-TGC growth through placental fatty acid oxidation. These results suggest a potential pathological mechanism of chemerin-related pregnancy complications and also imply the necessity to investigate further chemerin and lipid metabolism function in human trophoblast differentiation.

## Methods

### Animals care and treatment

The Committee approved animal welfare and experimental procedures for using Live Animals for Teaching and Research at Shenzhen Institutes of Advanced Technology, Chinese Academy of Sciences. The procedures were carried out in strict accordance with the related regulations. The experimental procedures were reviewed and approved by the Institutional Animal Care and Use Committees (IACUC) of Shenzhen Institutes of Advanced Technology (approval number: SIAT-IRB-170311-YYS-HUANGCHEN-A0330).

For the collection of serum and placental tissue from pregnant mice, 8-week-old female C57BL/6 mice (weight, 20–23 g) were mated with male C57BL/6 mice, and a copulatory plug was observed on day 0.5 of gestation. These mice were then anesthetized with isoflurane and sacrificed by cervical dislocation to collect serum and placentas.

For chemerin-treated mice, C57BL/6 pregnant mice were randomly divided into three groups: a control-saline group (*n* = 4), a chemerin 40 µg/kg group (*n* = 5), and a chemerin 60 µg/kg group (*n* = 5). The recombinant mouse chemerin peptide (2325-CM, R&D) was diluted in saline with 0.1% BSA solution and intravenously injected on E14.5 and E16.5. The chemerin concentration in E10-15.5 mice was estimated to be approximately 3 µg/ml based on the chemerin serum level in our data (Fig. 1A) and other studies [22, 38]. We calculated the total chemerin level in mouse serum using an estimated total serum volume of 2.0 mL and ensured that chemerin-treated concentrations were 0.4–0.8 times the serum chemerin level. The fetuses and placentas were collected and measured when the mice were sacrificed on E18.5.

### Western blot assay

Whole cells and tissue proteins were extracted using a lysis buffer (2% SDS, 50 mM Tris-HCI pH 7.6, 2 mM EDTA, and 10% glycerol). Proteins were quantified using the Pierce BCA Protein Assay Kit. Extracted total proteins were subjected to SDS-PAGE gel electrophoresis and transferred to a pure nitrocellulose blotting membrane (Millipore) after being blocked with 5% non-fat dried skim milk for 2 h at room temperature. The membrane was incubated with primary antibodies overnight at 4 ℃. The antibodies involved in this study are listed in detail in Additional file 1: Table S1. After washing with Tris-buffered saline with 0.1% Tween-20, horseradish peroxidase-conjugated secondary antibodies were incubated at room temperature for 1 h. The chemiluminescence analysis was performed using enhanced chemiluminescence (ECL; Bio-Rad Laboratories). Uncropped Western blots were in Additional file4.

### Elisa

The mice serum was collected from non-pregnant mice (*n* = 5), E5.5 (*n* = 6), E10.5 (*n* = 6), E15.5 (*n* = 6), E18.5 (*n* = 6), and postpartum 4 days (*n* = 4). Furthermore, the chemerin level was determined using a mouse chemerin ELISA assay (DY2325, R&D). Briefly, serum samples and chemerin standards were diluted with reagent diluent according to the detection range, and then diluted samples and standards were added to plates and incubated for 2 h. After washing the plates three times, they were incubated with the detection antibody for 2 h, followed by a repeated washing step and incubation with streptavidin-HRP antibody for 20 min. Finally, the density of the plates was determined using a microplate reader (Thermo) at 450 nm and corrected at 570 nm after reacting with substrate and stop solutions.

### Immunostaining and oil red O staining

Mouse placenta and gonadal fat tissue were fixed overnight at 4 °C in 4% PFA and embedded in paraffin. Five-micron sections were used for hematoxylin and eosin (H&E) staining. Immunostaining was performed on mouse placenta sections fixed with 4% PFA. The placenta tissue was sectioned at a thickness of 10 µm and blocked with 1% bovine serum albumin (BSA, Beyotime, ST023) for 1 h. The sections were sequentially incubated with primary antibodies overnight and corresponding secondary antibodies. The detailed antibody information is listed in Additional file 1: Table S2. The slides were placed under a coverslip with a mounting medium containing DAPI and examined under a fluorescence microscope (Olympus BX53, Japan). The placenta, liver, and gonadal fat tissue sections were immediately fixed in cold 4% PFA and sectioned at 5–7 µm for oil red O staining. After washing with distilled water, the slides were incubated in a 100% propylene glycol and 0.5% Oil Red O solution (O-1516; Sigma). Stained slides were washed with 85% propylene glycol solution and distilled water and mounted with glycerin.

### Flow cytometry

C57BL/6 pregnancy mice were sacrificed at E18.5 to collect the placentas in cold PBS. To obtain single-cell suspensions, the placentas were dissected and incubated in PBS containing 20% fetal bovine serum (FBS) and 1 mg/ml collagenase D (07902, StemCell). The tissue was digested in 37 °C incubators for 30 min and then passed through a 70-µm cell strainer. Erythrocytes in the placenta were lysed with red blood cell lysate buffer. Before staining, the cell suspensions were incubated with purified anti-CD16/32 (101,319, BioLegend) for 10 min on ice to block non-specific binding to Fc receptors. The antibody clones, fluorochromes, suppliers, and catalogue numbers in flow cytometry stains are listed in Additional file 1: Table S3. All FACS analyses were run using an FC500 flow cytometer (Beckman Coulter) and analyzed with FlowJo software.

### The spatiotemporal transcript expression of chemerin in placenta

The published stereo-seq dataset can be visualized by the interactive data portal (https://db.cngb.org/stomics/mpsta/). We downloaded the chemerin spatial expression data of E8.5 (E8.5_S1.MPSTA.h5ad, E8.5_S2.MPSTA.h5ad) and E14.5(E14.5_S1.MPSTA.h5ad, E14.5_S2.MPSTA.h5ad) for all clusters and generated a boxplot expression from E8.5 and E14.5 placental sections.

### Chemerin knockout mice generation and Experimental exposure in vivo

Chemerin knockout mice were obtained as a gift from Yangzhou University, which were produced by targeting the exon of murine chemerin with Crispr-Cas9 sgRNA (sequence: GAAATTAATACGACTCACTATAGGTGCACAATCAAACCAAACGGTTTTAGAGCTAGAAATAGC) and microinjected into fertilized eggs to create the knockout mouse [39]. We confirmed the absence of chemerin at the protein level in chemerin knockout mice. Chemerin knockout mice have not exhibited altered fertility or body weight compared to wild-type (WT) mice. WT and chemerin knockout pregnant mice were subjected to intraperitoneal injection with the control group (WT-ctrl, *n* = 4; chemerin KO-ctrl, *n* = 5) or etomoxir (WT-ET, *n* = 5; chemerin KO-ET, *n* = 6) at 20 mg/kg (HY-50202, MCE) on E14.5 and E16.5, respectively. Mice were anesthetized using isoflurane and sacrificed on E18.5; the placentas and embryos were weighed and then treated with 4% paraformaldehyde or frozen at − 80 °C for subsequent expression and analysis.

#### RNA-seq analysis gene expression and analysis

Transcript profiles were generated from wild-type (WT) placenta (*n* = 4, from 4 mice) and chemerin knockout placenta (*n* = 4, from 4 mice) at E 18.5. Total RNA was extracted using TRIzol reagent (Invitrogen, CA, USA), and RNA purity and integrity were assessed using a NanoDrop 2000 spectrophotometer (Thermo Scientific, USA) and an Agilent 2100 Bioanalyzer (Agilent Technologies, Santa Clara, CA, USA). Then, the DNA libraries were prepared with the Lumina Novaseq 6000 platform by OE Biotech, Inc., Shanghai, China. Transcript abundance was expressed as reads per kilobase of transcript per million mapped reads (RPKM), and a false discovery rate of 0.05 was used as a cutoff for significant differential expression. Statistical significance was determined through empirical analysis of digital gene expression, followed by the application of Bonferroni’s correction. Bioinformatic analysis was performed using the OECloud tools at https://cloud.oebiotech.com/task/. The Database for Annotation, Visualization, and Integrated Discovery (DAVID) was used to perform Gene Ontology (GO) functional and KEGG pathway enrichment analyses, with a p-value threshold of 0.05 considered statistically significant. The differentially expressed genes were listed in Additional file 2.

### Quantitative real-time PCR

Total RNA from WT (*n* = 4) and Chemerin KO placenta(*n* = 4) was extracted using the RNAiso Plus reagent (Takara, Shiga, Japan) and analyzed by quantitative real-time PCR (qRT-PCR) according to the manufacturer’s instructions (Revert RT Master and ReverTra qPCR RT Master Mix gDNA remover, Toyobo, Osaka, Japan). The primer sequences of mRNAs are presented in Additional file 1: Table S4. Gene expression levels were normalized to Gapdh using the ΔΔCT method, and the expression level was calculated for the WT group. Melt curve analysis for each primer set revealed only one peak for each product.

### Lipids and metabolic intermediate products detection

The placentas from chemerin-treated mice (ctrl, four mice; chemerin 40 µg/kg, five mice; 60 µg/kg, five mice) were homogenized in saline for protein quantification, and the collected supernatant was further examined for TG and CHO using the GPO-PAP colorimetric method. Briefly, placental TG and TC levels were measured using the triglyceride assay kit (A110-2–1, Nanjing Jiancheng Bioengineering Institute) and the total cholesterol assay kit (A111-2–1, Nanjing Jiancheng Bioengineering Institute), respectively, following the manufacturer’s instructions.

For the metabolic intermediate products test, the wild-type (WT) (5 mice) and chemerin knockout (KO) (5 mice) placental samples were analyzed using an Agilent 1290 Infinity chromatography system and an AB SCIEX QTRAP 5500 mass spectrometer for HPLC–MS/MS analysis. The placental tissue was taken in 40 mg quantities with each sample and homogenized with 200 µL of H2O. Then, 800 µL of a methanol/acetonitrile solution (1:1, v/v) and 10 µL of a 10 mmol/L SUCCINIC ACID-D6 internal standard solution were added to the mixture and subjected to ultrasound to precipitate the protein and freeze. The supernatant metabolites were injected for chromatographic separation using the 1290 Infinity UPLC (Agilent) and for mass spectrometry analysis with a 5500 QTRAP mass spectrometer (SCIEX). Analyses were determined by electrospray ionization using multiple reaction monitoring. Peak chromatographic area and retention time were analyzed with Multiquanta software. The standard substance of energy metabolites was used to calculate the retention time and identify metabolites. The detailed data for this experiment is in Additional file3.

The NADH and NADPH levels were further measured in WT (5 mice) and chemerin KO (5 mice) placental samples using the NAD +/NADH assay kit (#S0175, Beyotime, China) and the NADP +/NADPH assay kit (#S0179, Beyotime, China). Briefly, the placental tissues were incubated with extraction buffer for every 10 mg of tissue and disrupted cells with a homogenizer on ice, centrifuged at 12,000 × g for 5–10 min at 4 °C. Fifty to one hundred microliters of supernatant was moved into a tube followed by 30 min of incubation at 60 °C and then performed centrifugation at 10,000 g for 5 min at 4 °C. Twenty microliters of supernatant was moved into 96-well plates and incubated for 10 min at 37 °C, then followed by the addition of 10 µL color-developing solution. The NADH and NADPH values were measured by detecting the absorbance of the mixed liquid at 450 nm and were calculated using a standard value.

### Periodic acid-Schiff (PAS) staining

Placental paraffin sections were treated to remove paraffin, hydrated, and oxidized in 0.5% periodic acid solution for 5 min. Slides were placed in Schiff’s Reagent for 15 min, counterstained with hematoxylin, and washed in Scott’s tap water solution to enhance contrast. Sections were then dehydrated and mounted with Entellan mounting medium (EM Science) under glass coverslips. Consecutive sections of one placenta per genotype were examined.

### Chemerin shRNA lentiviral vector production

Mouse chemerin shRNA target sequences were synthesized and cloned into the pLV[shRNA]-EGFP:T2A: Puro-U6 vector (LV-GFP, Cyagen, China) to produce LV-chemerin-shRNA. These constructs were further transfected into packaging HEK293T cells as previously described to produce lentiviral particles [22]. The lentivirus-containing supernatant was then harvested and titrated using the QuickTiter Lentivirus quantitation kit (Cell Biolabs Inc., San Diego, CA, USA). For the chemerin target, we designed three target sequences and scrambled shRNA (Additional file1: Table S5) and assessed the target efficiency by measuring chemerin expression in lentiviral-infected islet endothelial cells (MS1 cells) (purchased from BeNa culture collection).

### Blastocysts transduction and trophoblast knockdown mouse construction

Mouse trophoblast-specific chemerin knockdown was achieved by lentivirus-mediated transduction into zona-free blastocysts as previously described [22]. First, 8-week-old CD-1 female mice (Beijing Vital River Laboratory Animal Technology Co., Ltd., Beijing, China) were mated with fertile or vasectomized males to produce pregnancy and pseudopregnancy. Second, the blastocysts were collected from E4 pregnancy mice and treated with acid Tyrode’s solution (Sigma–Aldrich, St. Louis, MI, USA) to remove the zona pellucidae. Individual zona-free blastocysts were incubated in 5 µl KSOM drops containing LV-scramble shRNA or LV-chemerin-shRNA (0.5 × 10^6^ transduction units/mL) for 6 h. Blastocysts were washed with M2 medium and transferred to E3 pseudopregnant mice (7–10 blastocysts/mouse). At E18, these mice were sacrificed to collect the placenta for further examination.

### Statistical analyses

All results are presented as the mean ± SEM of at least three independent experiments. Statistical significance calculations comparing two conditions were performed using a two-tailed unpaired Student’s *t*-test. Two additional data sets were analyzed using a one-way analysis of variance (ANOVA) with Tukey’s post hoc test. Two group variance analyses were performed using a two-way ANOVA and Tukey’s post hoc test. Statistical significance levels are calculated as follows: *p < 0.05; **p < 0.01; ***p < 0.001. These calculations were performed using GraphPad Prism 7 software (GraphPad, Prism Software).

## Supplementary Information

Additional file 1: Figures S1–S5 and Tables S1–S5.

Additional file 2. This Dataset described the differentially expressed genes (DEGs) between WT and Chemerin KO placentas with fold change > 2.0 and p-value threshold of 0.05.

Additional file 3. This dataset described the detailed information of metabolites identification and calculation in this study.

Additional file 4. This file provided the raw images for western blot in this study.

Additional file 5. This file provided the indivuiale value for each figure in main text and additional file.

## Data Availability

Availability of data All data generated or analyzed during this study are included in this published article, its supplementary information files and publicly available repositories. The information of the primers and antibodies used in this study were shown in Additional file 1. Differentially expressed genes (DEGs) between WT and Chemerin KO placentas (fold change > 2.0) are listed in Additional file 2. The mass spectrometry metabolites content in this study was detailed in an additional file 3. The raw images for western blot in this study are shown in Additional file4. We also provided the individual value data as additional file5 for each figure in main text and additional file. The dataset of the RNA sequencing in this study is available in the Gene Expression Omnibus (GEO) with GEO accession numbers (GSE298315) (URL: https://www.ncbi.nlm.nih.gov/geo/query/acc.cgi?acc=GSE298315). The metabolomics data have been deposited to MetaboLights repository with the study identifier MTBLS12552 (URL: https://www.ebi.ac.uk/metabolights/MTBLS12552). The spatial clustering data in the Fig. 1E and Additional file1: Fig. S2 including the raw data of E14.5(E14.5_S1.MPSTA.h5ad, E14.5_S2.MPSTA.h5ad) and E8.5 (E8.5_S1.MPSTA.h5ad, E8.5_S2.MPSTA.h5ad) could be downloaded for the restored web site (https://db.cngb.org/stomics/mpsta/download/).

## Abbreviations

CK8: Cytokeratin
EPC: Ectoplacental cone
P-TGC: Primary trophoblast giant cell
S-TGCs: Sinusoid trophoblast giant cells
TG: Triglyceride
TC: Cholesterol
KO: Knockout
WT: Wild type
SynT-I: Syncytiotrophoblast I cell
SynT-II: Syncytiotrophoblast II cell
SpT: Spongiotrophoblast cell
GlyT: Glycogen trophoblast cell
Tpbpα: Trophoblast-specific protein alpha
PAS: Periodic Acid-Schiff (PAS) staining
ACC: Acetyl-CoA carboxylase
P-ACC: Phosphorylation of ACC on serine site Ser79
ET: Etomoxir
CPT1A: Carnitine palmitoyl transferase I isoform
EVT: Extra-villous trophoblast
De: Decidua
Lab: Labyrinthine
Jz: Junctional zone
