# Supplementary material for: Chemerin sustains the growth of spongiotrophoblast and sinusoidal trophoblast giant cells through fatty acid oxidation
Source: BMC Biol. 2025 Jul 3;23:199. doi: 10.1186/s12915-025-02294-9 (PMC12226855; doi:10.1186/s12915-025-02294-9)
Supplement: Supplementary file 1 — Additional file 1: Figures S1–S5 and Tables S1–S5. [file 12915_2025_2294_MOESM1_ESM.docx]

**Additional File1**

**Table S1.** List of the antibodies for Western Blotting

| Antibody | Dilution | Vender | Catalog no. | RRID Portal |
| --- | --- | --- | --- | --- |
| Anti-Chemerin antibody | 1:1000 | abcam | ab103153 | AB_10861013 |
| Anti-Chemerin antibody | 1:1000 | proteintech | 10216-1 | AB_2269231 |
| Anti- CD36 antibody | 1:1000 | proteintech | 18836-1 | AB_10597244 |
| Anti- SREBP1 antibody | 1:1000 | abcam | ab28481 | AB_778069 |
| Anti- SREBP2 antibody | 1:1000 | abcam | ab30682 | AB_779079 |
| Anti- PPARγ antibody | 1:1000 | Cell Signaling Technology | #2435 | AB_2166051 |
| Anti- pACC1(Ser79) antibody | 1:1000 | Cell Signaling Technology | 3661s | AB_330337 |
| Anti- ACC1 antibody | 1:1000 | Cell Signaling Technology | 3676s | AB_2219397 |
| Anti- PPARD antibody | 1:1000 | Proteintech | 60193 | AB_10896827 |
| Anti- CPT1A antibody | 1:1000 | Proteintech | 15184-1-AP | AB_2084676 |
| Anti- ACTN antibody | 1:1000 | Cell Signaling Technology | #4970 | AB_2223172 |
| Anti-Tubulin antibody | 1:1000 | Proteintech | 10068-1-AP | AB_2303998 |
| Goat anti rabbit IgG H&L(HRP) | 1:5000 | Abcam | ab97051 | AB_10679369 |
| Goat anti mouse IgG H&L(HRP) | 1:5000 | abcam | ab6820 | AB_955438 |

**Table S2.** List of the antibodies for Immunostaining

| Antibody | Dilution | Vender | Catalog no. | RRID Portal |
| --- | --- | --- | --- | --- |
| Anti-Chemerin antibody | 1:400 | Santa cruz | sc-373797 | AB_10947246 |
| Anti- CD31 antibody | 1:400 | BD Biosciences Pharmingen | 550274 | AB_393571 |
| Anti- cytokeratin 8 antibody | 1:400 | TROMA-I | TROMA-I | AB_531826 |
| Anti- MCT1 antibody | 1:200 | Millipore-Sigma | AB1286-I | AB_90565 |
| Anti- MCT4 antibody | 1:200 | Millipore | ab3314P | AB_2286063 |
| Anti- laminin antibody | 1:400 | sigma | L9393 | AB_477163 |
| Anti- Tpbpα antibody | 1:200 | abcam | ab104401 | AB_10901888 |
| Rat IgG | 1:200 | Beyotime | A7031 |  |
| Chicken IgY | 1:200 | Beyotime | A7062 |  |
| Mouse IgG | 1:200 | Proteintech | B900620 | AB_2883054 |
| Rabbit IgG | 1:200 | Invitrogen | 31235 | AB_243593 |
| Alexa Fluor® 488 anti-rat | 1:400 | Abcam | ab150165 | AB_2650997 |
| Alexa Fluor® 568 anti-rabbit | 1:400 | abcam | ab175470 | AB_2783823 |
| Alexa Fluor® 568 anti-chicken | 1:400 | abcam | ab175711 | AB_2827757 |
| Alexa Fluor® 488 anti-mouse | 1:400 | abcam | ab150113 | AB_2576208 |

**Table S3.** List of the antibodies for flow cytometry

| Antibody | Dilution | Vender | Catalog no. | RRID Portal |
| --- | --- | --- | --- | --- |
| Anti-Chemerin antibody | 1:200 | Santa cruz | sc-373797 | AB_10947246 |
| Mouse IgG | 1:200 | Proteintech | B900620 | AB_2883054 |
| Alexa Fluor® 488 anti-mouse | 1:400 | abcam | ab150113 | AB_2576208 |
| PE-CD31 | 1:200 | BioLegend | 102507 | AB_312914 |
| Anti-mouse CD16/32 | 1:1000 | BioLegend | 101319 | AB_1574973 |

**Table S4.** List of the primer sequences of qPCR

| Gene | Forward | Reverse |
| --- | --- | --- |
| Awat1 | CCTCCTCAAGAAACGCAAAGG | CGTGAAGGTAGGGACCAGATC |
| Acss3 | AATGTCGCAAAGTAACAGGCG | GTGGGTCTTGTACTCACCACC |
| Ces2c | GCTGAATGCTGGGTTCTTCG | GCTGCCTTGGATCTGTCCTGT |
| Tpbpa | CAGAGAGTGGCGATGGGTTTT | GACAATGGCACAGTGGCTGTT |
| Prl7a2 | GGGAGAATGTGGCCTCTGTA | TTGAGCTTCGTCCAGGTTTT |
| Hand1 | GGGTTAAACCCGGTCTTTGG | AAGGACCTGCCGACCTCTTG |
| ASCL2 | CAGCTGCGAGGGAGAGCTAA | GATGCTCAGTAGCCCCCTAACC |
| Prl7b1 | GGGAGGACGTGGTCTCTGTA | TTTGGTGATTTGAGTGGCAA |
| Syna | CCTCACCTCCCAGGCCCCTC | GGCAGGGAGTTTGCCCACGA |
| Chemerin | TACAGGTGGCTCTGGAGGAGTTC | CTTCTCCCGTTTGGTTTGATTG |
| Gapdh | AGGTCGGTGTGAACGGATTTG | TGTAGACCATGTAGTTGAGGTCA |

**Table S5.** List of the shRNA sequences of Lentivirus（pLV[shRNA]-EGFP:T2A:Puro-U6>mRarres2[shRNA#3]）

| Gene | Target sequence |
| --- | --- |
| Scramble shRNA | CCTAAGGTTAAGTCGCCCTCG |
| {mRarres2[shRNA#1]} | TCAGGAGTTGCAATGCATTAA |
| {mRarres2[shRNA#3]} | CTTTGTGAGGTTGGAATTTAA |
| {mRarres2[shRNA#2]} | AGGGCCCTGAGAACCAAATAA |


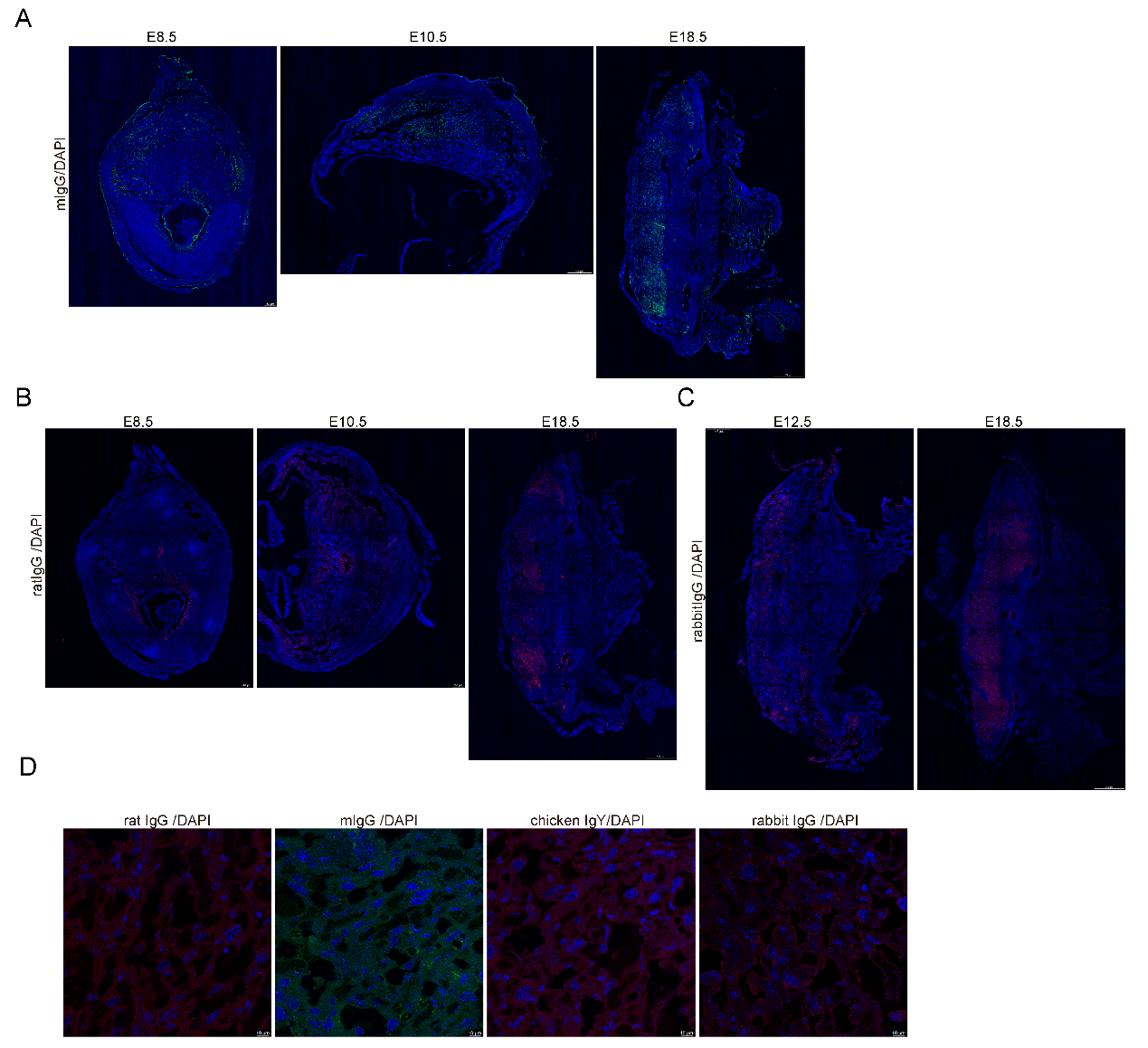


**FigureS1.Contrl IgG and IgY staining on different days of mouse placenta.**

**A** Mouse IgG as staining control on E8.5,10.5 and 18.5 placentas. **B** Rat IgG as staining control on E8.5,10.5 and 18.5 placentas. **C** Rabbit IgG as staining control on E12.5 and 18.5 placentas. **D** Mouse, Rat and rabbit IgG with chicken IgY staining on labyrinthine of E18.5 placenta.


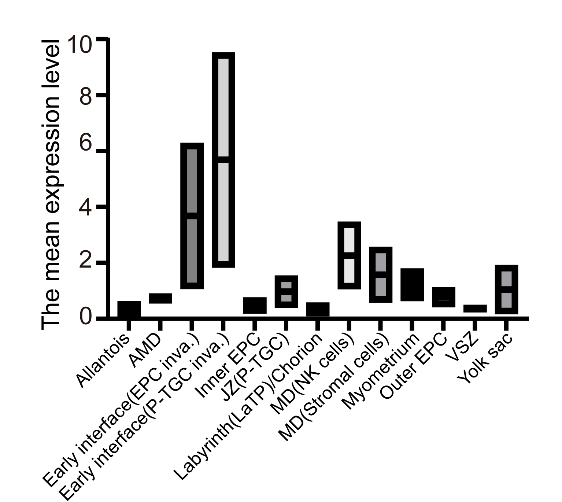


**FigureS2.chemerin’s spatiotemporal transcriptomic expression in the E8.5 placenta.**

The boxplot described the related mean expression of chemerin in the two E8.5 placenta section from the public spatiotemporal transcriptomic data (n=2). AMD (anti-mesometrial decidua); the early interface (EPC inva.); the early interface (P-TGC inva.); MD (mesometrial decidua), VSZ (vascular sinuses zone). The individual values of the expression of chemerin in two E8.5 placentas were showed in additional file 3.


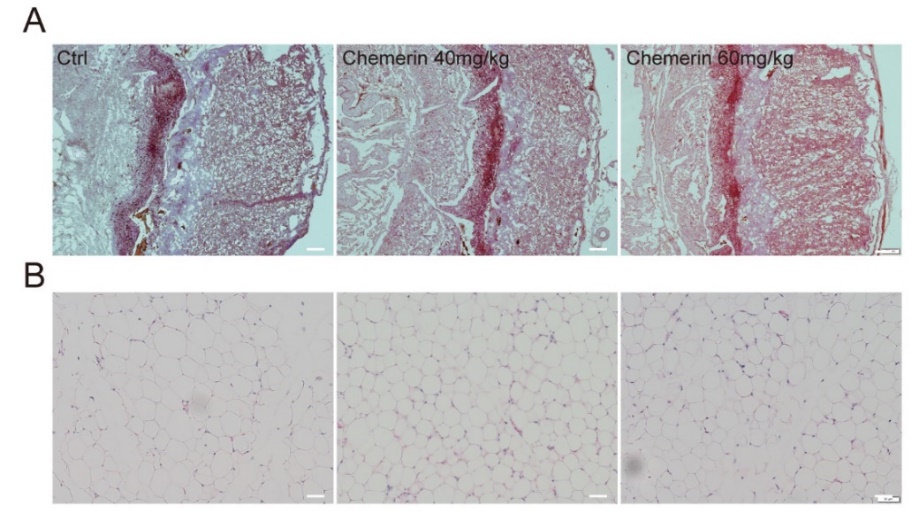


**FigureS3. excessive chemerin impairs placenta and fat lipid metabolism. (A).** Oil red O staining showed placenta lipid accumulation on placenta after chemerin treatment. (B). HE staining of gonadal fat tissue.


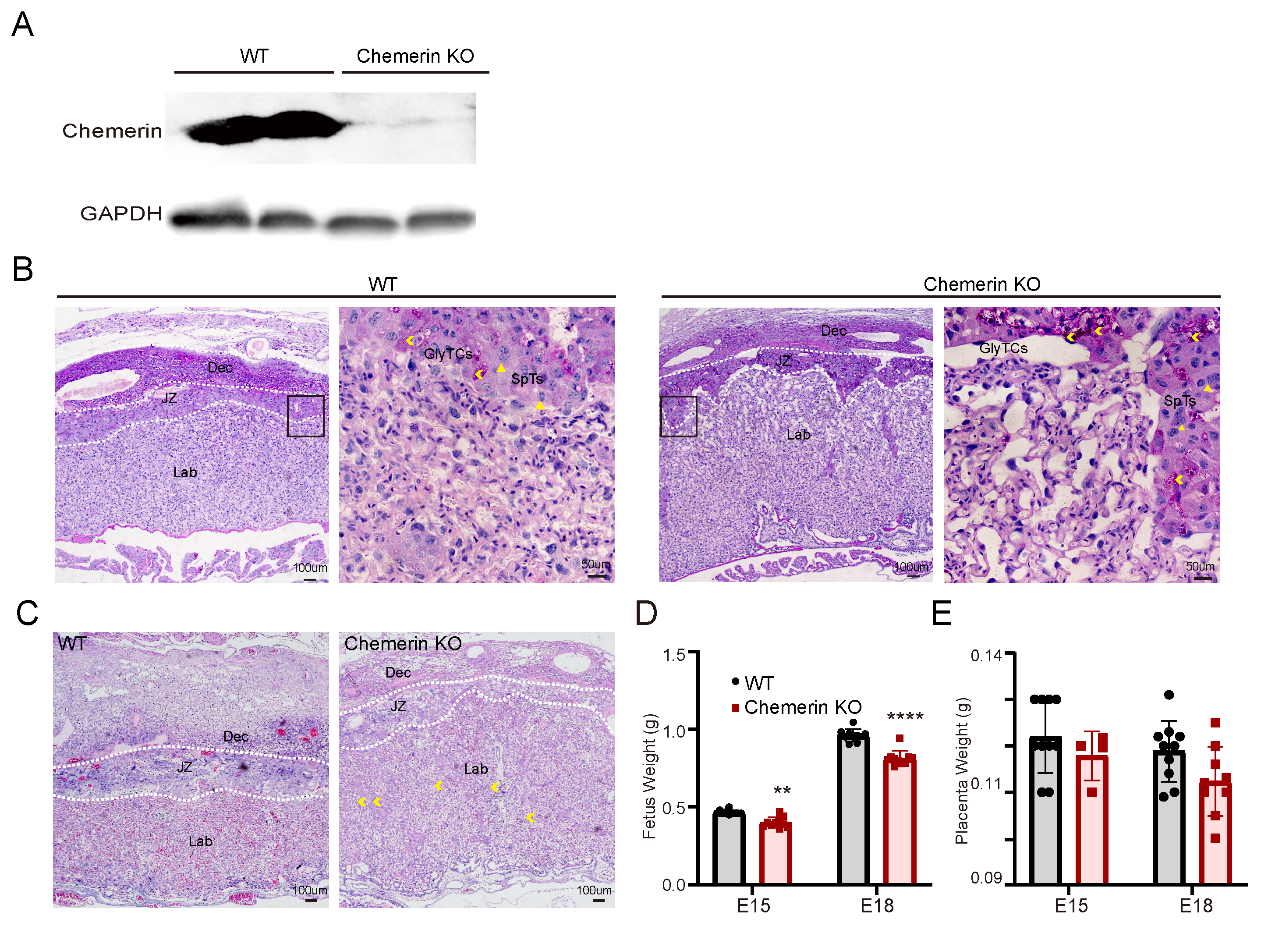


**FigureS4. Deficiency of chemerin exhibits abnormal glycogen trophoblast growth on placenta. A** Western blotting analysis for chemerin expression on WT and chemerin KO gonadal fat tissue. **B** Periodic Acid-Schiff (PAS) staining on WT and chemerin KO placenta and larger view on junction and labyrinthine area showed the growth of spongiotrophoblast cells (SpT) and glycogen trophoblast cells (GlyTC). **C** HE staining showed three layers on WT and placenta including decidua, Junction zone and labyrinthine. yellow arrow head indicated the area of loss fetal vessel. **D-E** Fetus weight and placenta weight at E15 and E18 were measured on WT and chemerin KO group (WT-E15.5, n=10, KO-E15.5, n=10; WT-E18.5, n=10, KO-E18.5, n=10). **p<0.01, ****<0.0001 compared with WT group with non-paired two-tailed *t* test. The individual values of the fetal weight (D) and the placenta weight (E) were showed in additional file 3.


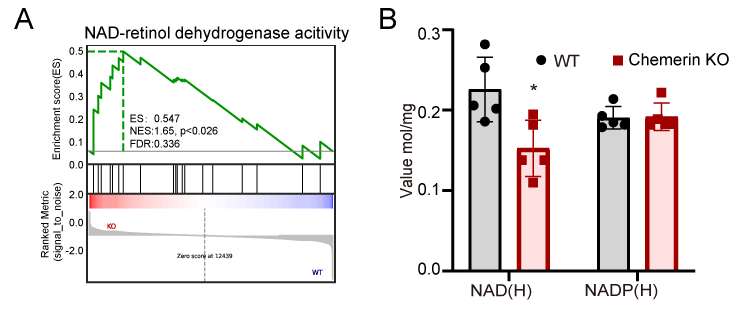


**FigureS5. Lack of chemerin on placenta results to decreased NAD(H) activity.**

**A** Enrichment plots of selected gene sets from GSEA showed that NAD-retinol dehydrogenase activity enriched on chemerin KO placenta. **B** The concentration of NAD(H) and NADP(H) on placenta from WT and chemerin KO mice, each group includes 5mice placenta. * p <0.05 and calculated NAD(H) and NADP(H) separately with non-paired two-tailed *t* test. The individual values of the NAD(H) and NADP(H) were showed in additional file 3.
