# Supplementary material for: Chemerin sustains the growth of spongiotrophoblast and sinusoidal trophoblast giant cells through fatty acid oxidation
Source: BMC Biol. 2025 Jul 3;23:199. doi: 10.1186/s12915-025-02294-9 (PMC12226855; doi:10.1186/s12915-025-02294-9)

**Additional file4_ Raw data for western blots.**

**Figure1**

Fig1.B

Chemerin,19KD:


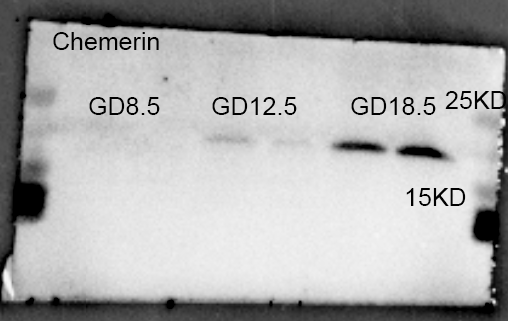


Tubulin,50KD


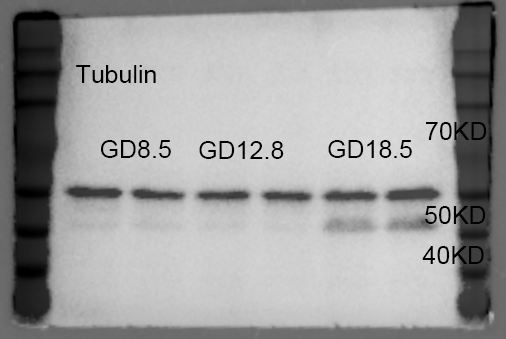


**Fig2.H**

CD36, 86KD


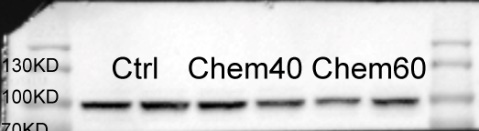


ACC,260KD


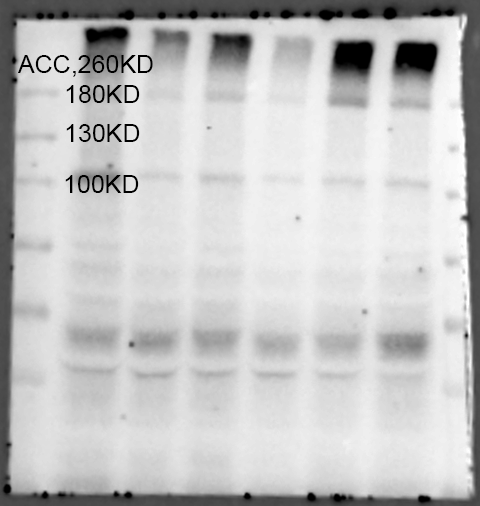


PPARγ,53KD


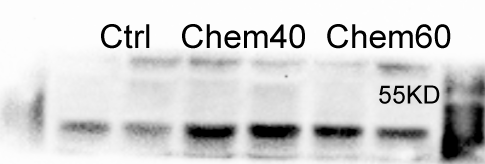


Srebp1,125KD


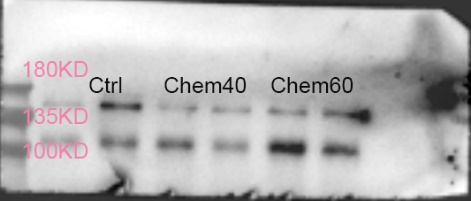


Srebp2,121KD


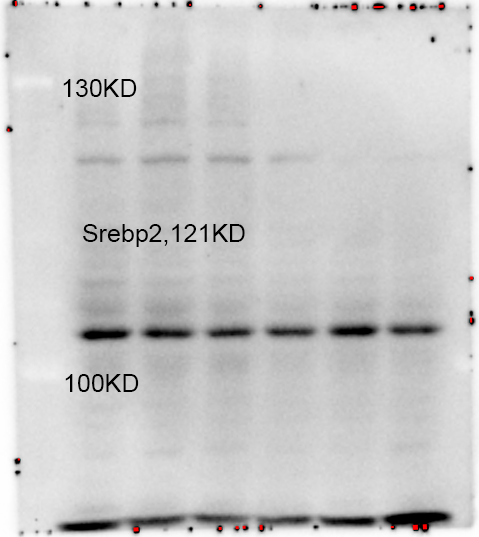


ACTN,42KD


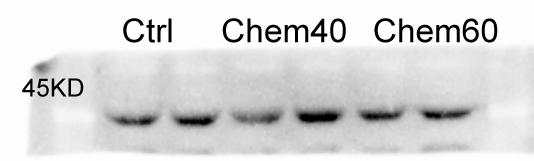


**Fig4.E**

CD36, 86KD


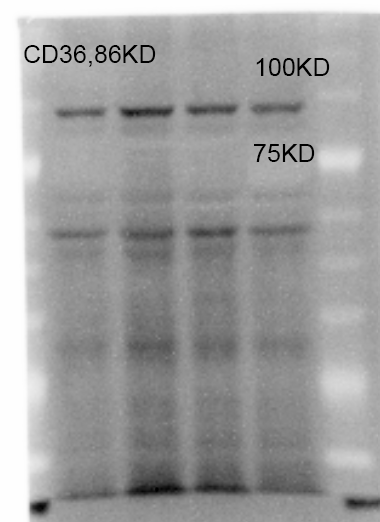


P-ACC,260KD


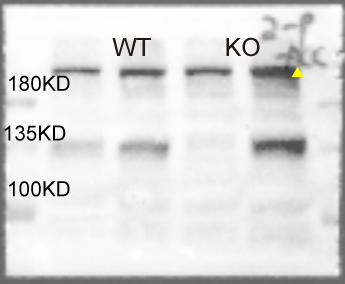


ACC,260KD


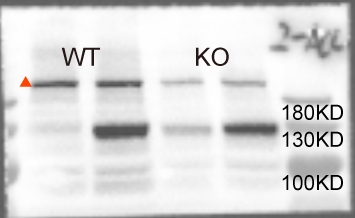


ACTN,42KD


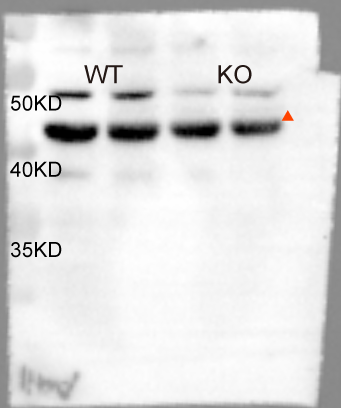


**Fig4.F**

PPARD,54KD


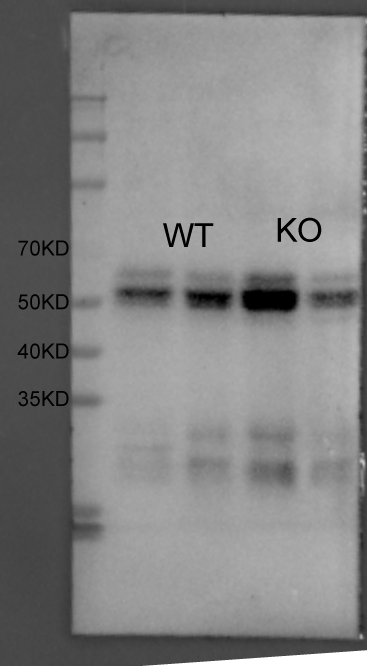


PPARα,52KD


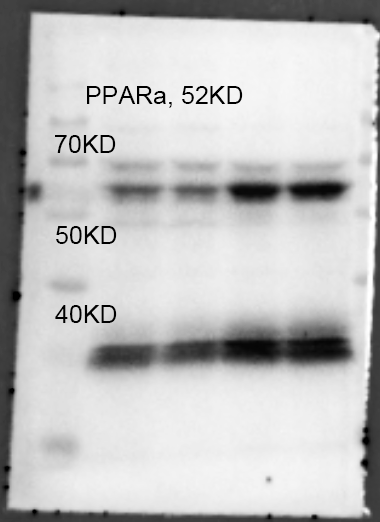


PGC1-α,110KD


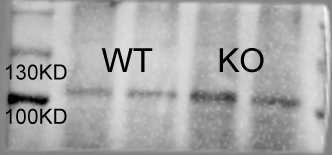


ACTN,42KD


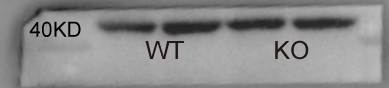


**Fig6.G**

CD36,86KD


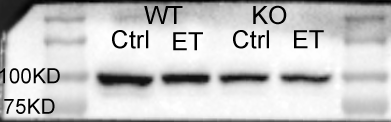


CPT1A,88KD


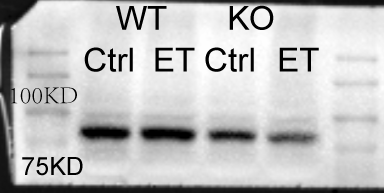


PPARγ,53KD


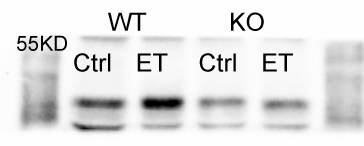


ACTN,42KD


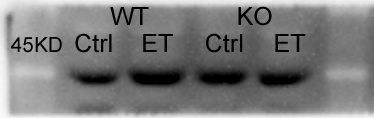


**Fig7.A**

chemerin,19KD


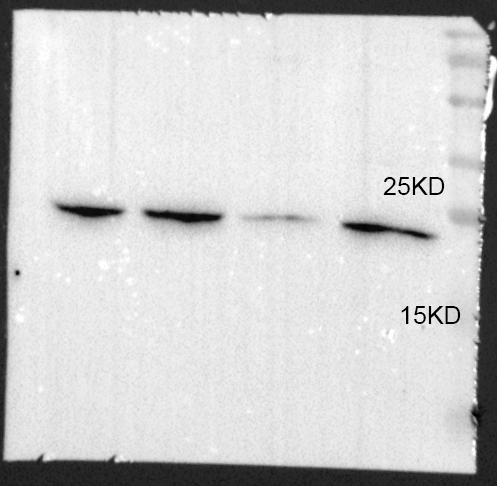


GAPDH,36KD


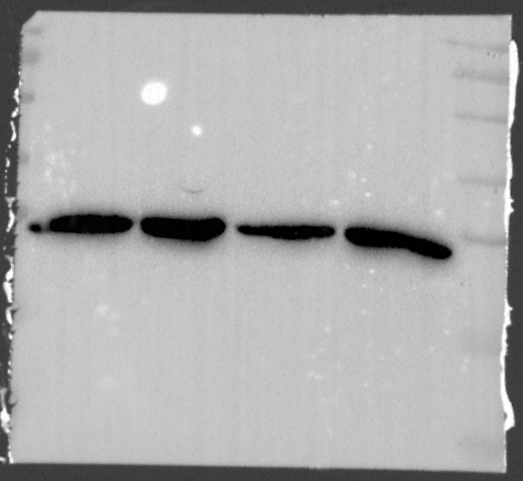


**Fig.S1**

Chemerin,19KD


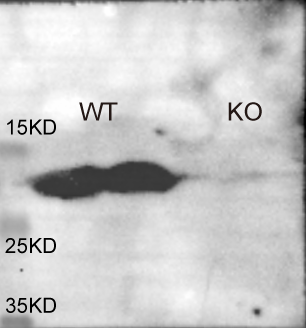


ACTN,42KD


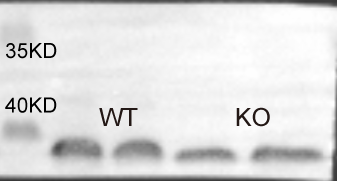

Supplement: Supplementary file 4 — Additional file 4. This file provided the raw images for western blot in this study. [file 12915_2025_2294_MOESM4_ESM.docx]
